# Determinants of longitudinal changes of CD4 cell count and survival time to death of HIV/AIDS patients treated at Yabelo General Hospital, the case of pastoralist area: Using joint modelling approach

**DOI:** 10.1371/journal.pone.0305519

**Published:** 2024-06-24

**Authors:** Galgalo Jaba Nura, Arero Biqicha Guyo, Markos Abiso Erango

**Affiliations:** 1 Department of Statistics, Arba Minch University, Arba Minch, Ethiopia; 2 Yabello General Hospital Coordinator, Borena Zone, Oromia Region, Ethiopia; Dilla University, ETHIOPIA

## Abstract

**Introduction:**

HIV/AIDS is a chronic disease that challenges public health worldwide and causes morbidity and mortality in humans. The main purpose of this study was to investigate the determinants of longitudinal changes in CD4 cell count and survival time to death among HIV/AIDS patients as adults from January 2016 to December 2019 at Yabelo General Hospital. The intellectual gap in this study was focused on the study area, which means that the study related to joint modeling doesn’t exist in the pastoralist community of Borena.

**Methods:**

This study involved 293 adult HIV-infected adults that could be collected from the recorded patient chart data, and the study design is a retrospective cohort design. The study used a Cox proportional hazard model, a linear mixed effect model, and a joint model, which is the combination of both model processes.

**Results:**

The joint model showed that longitudinal CD4 cell count is significantly associated with survival time (p-value = 0.0253). Covariates such as visiting time, age, weight, educational status, ART adherence, and functional status were statistically significant factors associated with mean changes in the CD4 cell count of HIV patients. WHO stage, educational status, place of residence, TB, family history, and opportunistic infection disease had a significant effect on the survival time of HIV patients.

**Conclusions:**

The estimated association parameter is a negative value, which indicates both outcomes are negatively associated, and higher values of the CD4 cell count are associated with better survival.

## Introduction

Human immunodeficiency virus (HIV) is a major public health issue around the world, and a lack of prevention programs leads to an increase in adults and infections among newborns, which are related to the morbidity and mortality of patients [1]. In HIV disease, immune function deteriorates over time on a continuum to AIDS, which is characterized by severe immune deterioration that is fatal if left untreated. By increasing CD4, antiretroviral therapy (ART) improves HIV-infected patients’ chances of survival [2].

Globally, the estimated numbers of HIV patients in 2021 there reach 38.4 million people living with HIV. In 2022, there have total of 39.9 million people living with HIV, 36.7 million adults living with HIV, 1.7 million children under the age of 15, and 1.5 million new infections [3]. Recent data show that progress has slowed and is uneven within and across countries. AIDS claimed the lives of 650,000 people in 2021, a 52% decrease from 1.4 million in 2010 and a 68% decrease from the peak of 2.0 million in 2004 [3]. Sub-Saharan Africa, which is home to two-thirds of all HIV patients globally, is the hardest-hit region, followed by Asia and the Pacific [3].

In Ethiopia, recently estimated numbers show a gradual decline in PLHIV, which fell from 612,925 in 2021 to 609,349 in 2022 [4], and mortality rates were high during the first year of ART treatment, with an estimated 20,000 PLHIV dying each year [5, 6]. In the Oromia region, from 2021 to 2022, there was a gradual decline, with 157,198 to 156,567 people living with HIV/AIDS being assigned under the prevalence reported [4]. The researcher intended to investigate the determinants of longitudinal changes in CD4 cell count and survival time to death among adults’ HIV patients by using a joint model from January 2016 to December 2019 at Yabelo General Hospital.

HIV/AIDS is a chronic, untreatable cure disease that has created unique challenges for physicians and health-care systems around the world. HIV/AIDS is an epidemic disease that affects not only individuals’ health but also households, communities, and nations’ development and economic growth. Those at risk of HIV continue to lack access to prevention, care, and treatment, and there is no cure [7]. It will help bring HIV patients to the attention of public health policymakers, researchers, and the general public, as well as assist those of us who work on aspects of care, support, and treatment for HIV/AIDS patients. The results of this study will also provide information about the factors that predict changes in CD4 cell count and survival time in HIV-infected patients.

### Data and methodology

#### Study area, design and period

This study was conducted at the Yabelo General Hospital, which is located in the pastoralist area of Yabelo town, Borena Zone. The study design is a retrospective cohort study since all of the events and exposures reported on the review subjects’ patient cards and information sheets occurred in the past. Patients were tested at Yabelo General Hospital from January 2016 to December 2019. The study was carried out after getting approval for data collection from the ethical committee at Yabelo General Hospital. In addition, all study methods were performed based on relevant guidelines and regulations laid down by the Committee.

#### Eligibility criteria

Based on the inclusion and exclusion criteria, 293 adult patients were selected from the patients’ medical cards. This study was focused on HIV-infected patients under follow-up ART treatment from January 2016 to December 2019. This study included all adult HIV-positive patients attending at least a minimum of three visits for longitudinal response, adult patients whose age is greater than or equal to 15 years, and patients who have started treatment within the treatment follow-up study period between January 2016 and December 2019.

Patients who fulfilled all variables of interest and whose information included the diagnosis of medical conditions were included in the study. Patients whose medical charts were incomplete, medical charts were not found, patients were dropped out, transferred to another place, recovered or restarted, and were out of the study period were excluded from this study.

#### Variables in the study

The response variables considered in the study were the CD4 cell count and survival time of the HIV patient under ART follow-up. In this study, several predictors were considered for both survival and longitudinal cases. Explanatory variables included in this study are sex, age, marital status, educational status, weight, place of residence, WHO stages, TB, visit time, adherence to ART treatment, functional status, specimen type, religion, family history, and opportunistic infection disease.

#### Method of analysis

Before modeling, it can perform exploratory data analysis to study various structures and patterns displayed in the data collection. This entails gathering summary data such as frequencies and percentages in a specific group. Individual profile plots, mean structure plots, and variance structure plots were also investigated in order to get some insights into the data [8].

#### Survival analysis model

The proportional hazard model was used as the basic model for survival data in this investigation. Survival analysis is a field of statistics that examines the expected period of time until one or more events occur, such as death in biological organisms or failure in mechanical systems [9]. The Cox proportional hazards model implies that the hazard function (t, X) is connected to the covariates as a product of a baseline hazard and a function of covariates, as shown in the form of the equation below.


$$\lambda_{i}(t|X_{i})=\lambda_{0}(t)\exp\left({X_{1i}}^{T}\beta_{1i}\right)$$

$$=\lambda_{0}(t)\exp\left(\beta_{11}X_{11}+\beta_{12}X_{12}+\cdots +\beta_{1p}X_{1p}\right)$$ (1)

Where, λ_0_(t) is the baseline hazard function, $X_{1}={\left(X_{11},\ldots ,X_{1p}\right)}^{\prime }$ is a set of covariates from i^th^ patients and $\beta_{1}=(\beta_{11},\beta_{12},\beta_{13},\ldots ,\beta_{1p})'$ is unknown p regression parameters which measure the effect of the covariates on the risk of death.

#### Longitudinal models

A linear mixed model (LMM) is a parametric linear model that quantifies the associations between a continuous dependent variable and several predictor factors for clustered, longitudinal, or repeated measurement data [10]. In this study, the CD4 cell count measurements would have been considered as longitudinal data on the response variable taken from the same HIV patients over repeated observation or visit times. Generally, the LMM formula for longitudinal endpoints has the form in the below equations.


$$Y_{i}=X_{i}\beta +Z_{i}u_{i}+\varepsilon_{i}$$

$$\left\{ \begin{array}{c} m_{i}(t)=X_{i}\beta +Z_{i}u_{i}\\ u_{i}∼N(0,G)\\ \varepsilon_{i}∼N(0,R)\\ b_{1},\ldots b_{n},\;\mathrm{and}\;\varepsilon_{1}\ldots \ldots \varepsilon_{n}.\;are\;independent \end{array}\right.$$ (2)

Where, *Y*_*i*_ is the corresponding true underlying longitudinal measures CD4 cell count of the i^th^ subject; *X*_*i*_ and *Z*_*i*_ were the *n*_*i*_ *x p* and *n*_*i*_ *x k* design matrix of fixed and random effects, respectively. The *p x 1* and *k x 1* vectors of corresponding fixed and random effects parameters are *β* and *u*_*i*_ respectively and *ε*_i_ is *n*_*i*_ *x 1* vector of the measurement error it distributed as *N (0*, *R)* is a vector of residuals components. Then, the random effects (*u*_*i*_) is distributed as *N*(0,***G***), which are assumed to be normally distributed with mean zero and variance-covariance matrix **G** and independently of each other. The covariance of *u*_*i*_ and *ε*_i_ are zero (i.e., Cov(ui, *ε*_i_) = 0). Furthermore *R = σ*^2^*I*_*ni*_ is the *n*_*i X*_ *n*_*i*_ positive–definite variance covariance matrix for the errors in subject *i*, and *I*_*ni*_ denotes the *n*_*i X*_ *n*_*i*_ identity matrix.

#### Joint longitudinal and survival model

The longitudinal and survival processes are expected to be conditionally independent, given unobserved random influences in the joint model. The random effect explains both the link between the longitudinal and event outcomes as well as the correlation between repeated measurements in the longitudinal process. Because both processes share this random effect, this form of joint model is referred to as a shared parameter model [11]. Then the joint model that links the longitudinal response to the time-to-event process through current value parameterization has the form of the equation below:

$$\lambda_{i}(t|M_{i}(t),x_{i})=\lambda_{o}(t)\exp\left(\beta^{T}X_{i}+\delta m_{i}(t)),t>0(3\right)$$ (3)

Where,

M_i_(t) = {m_i_(s), 0 ≤ s < t} denotes the history of the true unobserved longitudinal process up to time point t given in Eq (2), *λ*_*o*_*(t)* denotes the baseline risk function, and X_i_ is a set of baseline covariates with a corresponding vector of regression coefficients *β* and *δ* is association parameter which quantifies the effect of the underlying longitudinal outcomes to the risk for an event.

### Ethical approval and consent

The study was carried out after getting permission from the Statistics Department at Arba Minch University. In this regard, the official letter of cooperation referenced in stat/623/2015 was written to the ethical approval committee at Yabelo General Hospital. Then, the ethical committee approved the letter and gave permission to collect data from patients’ records and use it in the study. For the purpose of confidentiality, there were no links to individual patients, and all data had no personal identifier. Therefore, informed consent from the patient has been waived by the Yabelo General Hospital ethical committee.

## Results and discussions

### Descriptive statistics

Out of 293 HIV/AIDS patients eligible for the study, 179 (61.1%) were females, and the remaining 114 (38.9%) were males. Among those females, 33 (18.4%) were dead, and the remaining were censored. Of the male patients, 36 (31.6%) were dead, and the remaining others were censored. Among the entire subjects integrated in this study, 146 (49.8%) of the patients were not formal educated, 84 (28.7%) of the patients were at the primary level, 37 (12.6%) of the patients were at the secondary educational level, and 26 (8.9%) of the patients were at the tertiary level. Out of those entire educational levels, the death proportion of patients was 45 (30.8%), 13 (15.5%), 4 (10.8%), and 7 (26.9%) who were not educated at any level, at the primary, secondary, and tertiary levels, respectively.

Out of the total samples, 210 (71.7%) patients had no tuberculosis (TB), and 83 (28.3%) had tuberculosis disease. Out of the no-tuberculosis (TB) patients, 35 (16.7%) died, and 34 (41.0%) of the patients who had tuberculosis (TB) died. There were 221 (75.4%) of the patients who had worked, 27 (9.2%) were bedridden, and 45 (15.4%) patients came in with the ambulatory. Out of the working status, 50 (22.6%) patients died; from the bedridden, 7 (25.9%) patients died, and 12 (26.7%) died from ambulatory patients (see Table 1).

**Table 1:** Baseline categorical variables with survival status of HIV/AIDS patients in Yabelo General Hospital from January 2016 to December 2019.

Covariates	Censoring Status		Number Out of Total (%)
	Censored (%)	Death (%)	
**Sex**			
Male	78(68.4)	36(31.6)	114(38.9)
Female	146 (81.6)	33 (18.4)	179(61.1)
**Marital Status**			
Single	29 (69.0)	13 (31.0)	42(14.3)
Married	105 (75.5)	34 (24.5)	139(47.4)
Widow	55 (85.9)	9 (14.1)	64(21.8)
Divorced	35 (72.9)	13(27.1)	48(16.4)
** Educational**			
Not Formal	101 (69.2)	45 (30.8)	146 (49.8)
Primary	71 (84.5)	13 (15.5)	84 (28.7)
Secondary	33 (89.2)	4(10.8)	37 (12.6)
Tertiary	19 (73.1)	7(26.9)	26 (8.9)
**WHO Stage**			
Stage I	125 (77.2)	37 (22.8)	162 (55.3)
Stage II	31 (86.1)	5 (13.9)	36 (12.3)
Stage III	57 (75.0)	19 (25.0)	76 (25.9)
Stage IV	11 (57.9)	8 (42.1)	19 (6.5)
**Residence**			
Urban	147 (84.5)	27 (15.5)	174 (59.4)
Rural	77 (64.7)	42 (35.3)	119 (40.6)
**TB**			
No	175 (83.3)	35 (16.7)	210 (71.7)
Yes	49 (59.0)	34 (41.0)	83 (28.3)
**Adherence**			
Good	142 (77.2)	42 (22.8)	184 (62.8)
Poor	64 (77.1)	19(22.9)	83 (28.3)
Fair	18 (69.2)	8 (30.8)	26 (8.9)
**Functional Status**			
Ambulatory	33 (73.3)	12 (26.7)	45 (15.4)
Bedridden	20 (74.1)	7 (25.9)	27 (9.2)
Working	171 (77.4)	50 (22.6)	221 (75.4)
**Specimen Type**			
Whole type	44 (77.2)	13 (22.8)	57 (19.5)
Plasma	154 (75.9)	49 (24.1)	203 (69.3)
DBS	26 (78.8)	7(21.2)	33 (11.3)
**Family History**			
No	158 (83.2)	32 (16.8)	190 (64.8)
Yes	66 (64.1)	37 (35.9)	103 (35.2)
**Opportunistic Infections Disease**			
No	167(83.1)	34(16.9)	201 (68.6)
Yes	57(62.0)	35(38.0)	92 (31.4)
**Religion**			
Protestant	34(75.6)	11(24.4)	45 (15.4)
Orthodox	73(70.2)	31(29.8)	104 (35.5)
Waaqeffataa	62(83.8)	12(16.2)	74 (25.3)
Muslim	55(78.6)	15(21.4)	70 23.9)

### Descriptive statistics of longitudinal data

Table 2 shows the result of the summary statistics of longitudinal CD4 cell count per mm^3^ across visiting time groups. The number of participants’ counts every six months after baseline diagnosis; the mean and standard deviations are based on each visiting time group. The sample size of participants varied between visiting time groups: 293 (100%), 293 (100%), 293 (100%), 252 (86.0%), 229 (78.16%), 195 (66.55%), 156 (53.24%), 111 (37.88%), and 54 (18.43%), which indicates the first (baseline) up to the ninth visiting time period, respectively. The number of participants between follow-up periods was decreasing over visiting times (in months) due to different reasons, including death, a limiting interval of study, and others.

**Table 2:** Summary statistics of the longitudinal CD4 cell count of HIV/AIDS adult patients across visiting time groups.

**Visiting Time**	**Baseline**	**1**^**st**^ **visit**	**2**^**nd**^ **visit**	**3**^**rd**^ **visit**	**4**^**th**^ **visit**
**Number (%)**	293 (100)	293 (100)	293 (100)	252 (86.0)	229 (78.16)
**Mean**	485.23	531.74	554.42	564.43	575.82
**SD**	251.27	261.19	260.27	260.17	248.38
**Visiting Time**	**5**^**th**^ **visit**	**6**^**th**^ **visit**	**7**^**th**^ **visit**	**8**^**th**^ **visit**	
**Number (%)**	195 (66.55)	156 (53.24)	111 (37.9)	54 (18.4)	
**Mean**	594.81	606.74	663.15	664.50	
**SD**	260.88	261.64	259.15	289.02	

The mean CD4 cell count per mm^3^ of adult patients increased with an increasing rate of visit time until the end of periods. The baseline mean CD4 cell count was 485.23 per mm3, and the standard deviation was 251.27 CD4 cells per mm^3^, implying that the first diagnosis (baseline) of adults was at higher risk than other periods but in the second, lower range of variations from all period groups.

#### Exploring of individual profile plot

**Fig 1:**
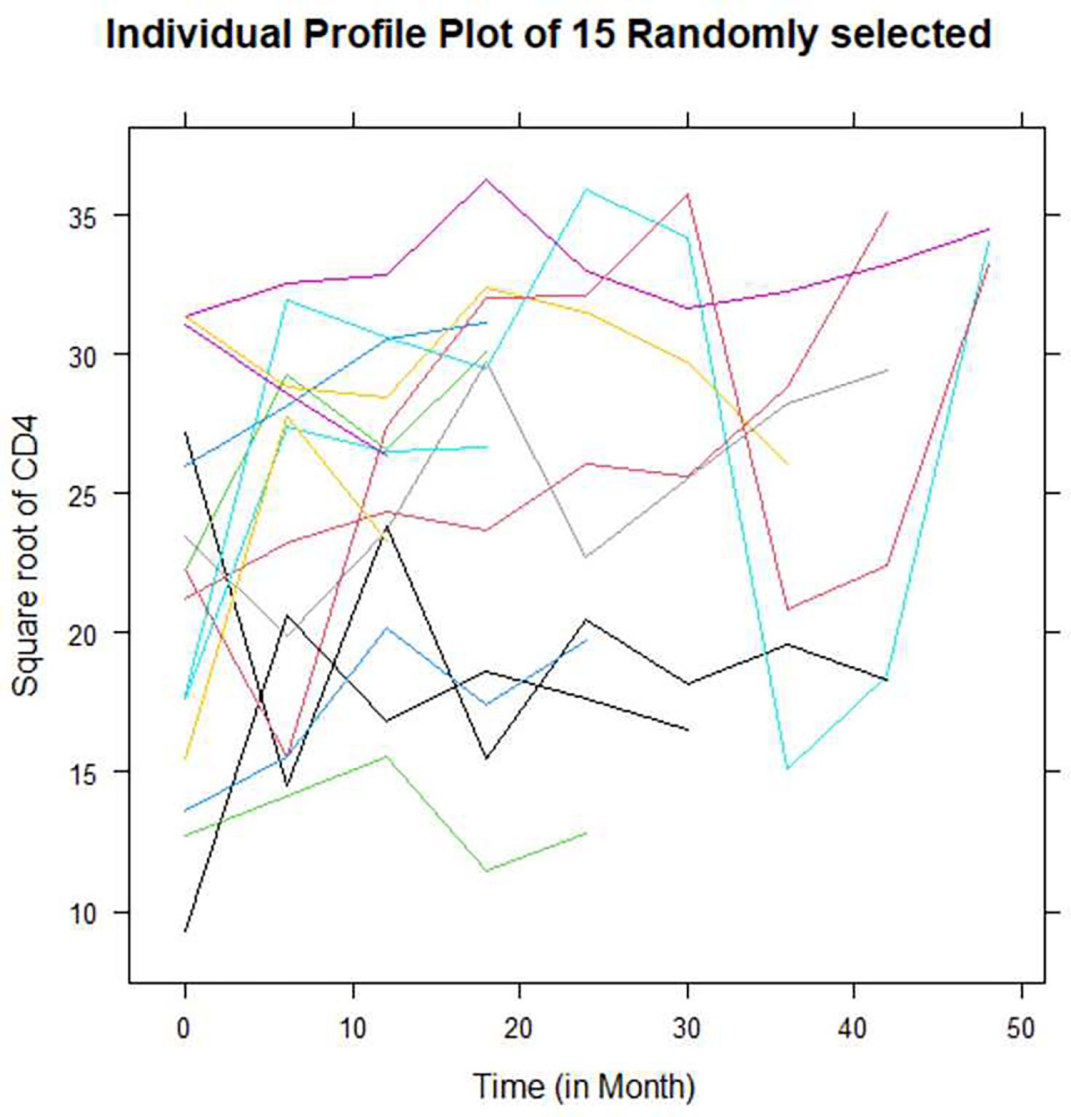
Individual profile plot of fifteen randomly selected HIV/AIDS patients.

Fig 1 demonstrates an individual profile plot of the longitudinal square root of the CD4 cell count of fifteen randomly selected from the total of 293 HIV/AIDS-infected adult patients over time, with some trajectories being steeper while others were almost horizontal, indicating the possible variability in the slope and intercept of square root CD4 cell counts.

The plot displayed information on variability between square root CD4 cell counts and shows that there is a change in CD4 cell count over time. Some values of CD4 cell counts increase with increasing time, while others decrease over time. It appears that there is a fluctuation in CD4 cell count over time after they initiated ART, and the variability of CD4 cell count seemed lower at the beginning and larger at the end.

#### Random effects selection and model diagnostics

The assumptions of the two models have been assessed using separately modeled longitudinal and survival data. Examining the assumptions of the survival models, the Schoenfeld residuals and the GLOBAL test with (Chi-square value = 9.5634, df = 14, P-value<0.79) were considered; the outcome shows that the reveal assumption was met (see S1 Fig). The normality assumption of the linear mixed effect model was checked using a normal Q-Q plot and histogram. When we have plots, the histogram and real CD4 count demonstrate that the normality assumption has been violated. The square root transformation method that was used on the original CD4 cell count data can be used to address the normality problem. The result showed that the assumption was satisfied (See the S2 Fig).

In linear mixed effect model, it is essential to fix the random effect to be included in the final model. After the computed the random intercept, slope, and both, then we check that the AIC, BIC and log likelihood have a lower value. So, the inclusion of random intercept and random slope was used in the linear mixed effect model, which is appropriate for the final model of square root CD4 count over time(see Table 3).

**Table 3:** Selection of random effects for linear mixed effects model.

Random effects	AIC	BIC	-2Loglik
Random intercept	11235	11257	11232.12
Random slope	11579	11602	11576.59
Random intercept and random	11222	11255	11214.82

#### Joint model for longitudinal and time to event

The joint modeling approach was employed in the following models: the cox proportional hazard model and the linear mixed effect model. Covariates such as visiting time, age, weight, educational status, ART adherence, and functional status were found to be statistically significant and associated with mean changes in the longitudinal CD4 cell count of HIV/AIDS adult patients. Also, variables having a statistically significant effect on the survival time of HIV/AIDS patients were WHO stage, educational status, place of residence, TB, family history, and opportunistic infection disease. Being of educational status was common and significant for both sub-models.

According to our study with the joint model, the mean changes of the square root of the CD4 cell count were positively correlated with visiting time (estimate mean = 0.069, 95% credible interval: [0.0531, 0.0859]), that is, the changes in the CD4 cell count increase significantly by a factor of 0.069 each month, and a 95% credible interval suggests that the interval contains the estimated mean value.

The mean change of the square root of the CD4 cell count was significantly lower with age, and CD4 cell counts decreased when the age was increased by a year. A unit increase in the body weight of the patients following treatment increases the mean changes in the CD4 cell count by 0.069 when keeping the other covariates constant. When the influence of the other variables was kept constant, the mean changes in the square root of the CD4 cell count were 1.419 times higher for patients with secondary education compared to illiterate patients.

Concerning ART adherence, the estimated mean changes of the square root of CD4 cell count were 2.179 times higher for those who have good adherence compared to the fair stages and 2.312 times higher for poor adherence compared to the fair stages, respectively, for the controlling effect influence variables. Regarding functional status, the mean change in the square root of CD4 cell count was 2.478 times higher for bedridden compared to ambulatory when controlling for the other variable (see Table 4).

**Table 4:** Parameter estimate of joint modeling of longitudinal and survival outcomes.

**Longitudinal sub-model**				
**Variables**	**Estimate**	**SE**	**[95% CI]**	**P-value**
Intercept	17.964	1.553	[14.8758, 20.9969]	0.000
**Age**	-0.046	0.02	[-0.0855, -0.0066]	0.0237
**Weight**	0.069	0.017	[0.0346, 0.1034]	0.000
**Educational (**Not educated^**®**^**)**				
Primary	-0.016	0.578	[-1.1268, 1.1178]	0.9753
Secondary	1.419	0.733	[0.0096, 2.8331]	0.0487
Tertiary	-1.366	0.854	[-3.0463, 0.3470]	0.109
**WHO Stage (**Stage **I**^**®**^**)**				
Stage II	-0.177	0.727	[-1.5877, 1.2331]	0.805
Stage III	-0.419	0.608	[-1.6105, 0.7700]	0.4783
Stage IV	-0.829	1.069	[-2.9546, 1.2840]	0.432
**ART Adherence (**Fair^**®**^**)**				
Good	2.179	0.952	[0.3157, 4.0797]	0.0227
Poor	2.312	0.956	[0.4259, 4.1483]	0.0167
**Opportunistic infection (**No^**®**^**)**				
Yes	-0.594	0.535	[-1.6332, 0.4551]	0.2647
**Functional (**Ambulatory^**®**^**)**				
Bedridden	2.478	0.937	[0.6651, 4.3516]	0.0100
Working	-0.359	0.698	[-1.7310, 1.0222]	0.6010
**Visiting Time**	0.069	0.008	[0.0531, 0.0859]	0.000
**Survival Sub-model**				
**Variables**	**Estimate**	**SE**	**[95% CI]**	**P-value**
**Sex (**Female^**®**^**)**	0.504	0.427	[-0.3226, 1.3571]	0.2383
**Marital Status** (Divorced^®^)				
Married	-0.384	0.365	[-1.1333, 0.3442]	0.2990
Single	0.365	0.408	[-0.4520, 1.1548]	0.3670
Widow	-0.703	0.454	[-1.5873, 0.2179]	0.1147
**WHO (**Stage I^**®**^**)**				
Stage II	-1.119	0.538	[-2.2789, -0.2159]	0.0117
Stage III	0.001	0.319	[-0.6541, 0.6115]	0.9817
Stage IV	-0.457	0.421	[-1.3190, 0.3532)	0.2643
**Educational** (Not educated^®^)				
Primary	-0.565	0.324	[-1.2321, 0.0473]	0.0720
Secondary	-1.178	0.574	[-2.4172, -0.1973]	0.0153
Tertiary	-0.446	0.434	[-1.3338, 0.3409]	0.3130
**Place of Residence (**Rural^**®**^**)**				
Yes	-0.882	0.266	[-1.3883, 0.3741]	0.0043
**TB (**No^**®**^**)**				
Yes	0.562	0.267	[0.0532, 1.0853]	0.0293
**Family History (**No^**®**^**)**				
Yes	0.5800	0.2631	[0.0700, 1.0791]	0.0310
**Opportunistic infection (**No^**®**^**)**				
Yes	0.7980	0.2736	[0.2647, 1.3141]	0.0013
**Sigma**	4.1014	0.0759	[3.9568, 4.2550]	0.000
**Associations (*α*)**	-0.1150	0.0482	[-0.2092, -0.0184]	0.0253
**Variance components**				
**Random Coefficients**	**Standard deviation**			
Intercept(*σ*_*o*_^2^)	3.6438			
Time(*σ*_*b*_^2^)	0.0617			
Corr(*σ*_*o*_^2^, *σ*_*b*_^2^)	-0.3952			

In the survival sub-model, the estimated hazard ratio of death for HIV-infected adults in clinical stage II compared to HIV/AIDS-infected adult patients in clinical stage I was exp (-1.119) = 0.3265, indicating that the risk of death for the HIV-infected adult in clinical stage II was 67.35% (p-value = 0.0117) times lower than for the HIV-infected adult in clinical stage I when all other variables were held constant.

The estimated hazard ratio of secondary educated level patients was exp (-1.178) = 0.3078, which means that the risk of death of the HIV/AIDS-infected adult who had been educated at the secondary level was 69.22% (p-value = 0.0153) times lower than that of those who had not attended school when keeping the other variables. The HIV/AIDS adult patients who lived in urban areas had a lower risk of death than those who lived in rural areas.

The relationship between survival time and the mean change in square CD4 cell count was parameterized by subject-specific deviation from the intercept and the overall linear slope of visiting time. The association value (a) was significantly different from zero, showing that the square root of CD4 cell counts is strongly associated with the probability of mortality. The association parameter’s estimated negative value (-0.1150) suggested that the slope of the square root of CD4 counts was negatively linked with the risk of death and that a unit increase in the square root of the CD4 count decreased the chance of death.

## Discussions

This study attempted to assess explanatory variables that are associated with longitudinal CD4 cell count and the survival time of adults due to HIV in Yabelo General Hospital. The longitudinal measurement of the mean change of the square root of the CD4 cell count and its association with the survival time of HIV/AIDS patients was explored using a joint model.

The findings of this study revealed that patients with a secondary education had a lower chance of death than those with no formal education at all. The current finding is consistent with previous findings from a similar study [12, 13], indicating that there are significant effects on the survival sub-model time of adult patients.

Visiting time has been found to have a significant effect on the mean changes in the square root of the CD4 cell count of patients. This study recognized that visiting time was positively correlated with the mean change of the square root of the CD4 cell count and increased significantly by a factor of 0.069 in each month. This result is supported by the studies conducted on HIV/AIDS patients by [14].

ART adherence status is a statistically significant factor for mean changes in the square root of the CD4 cell count in adult HIV-positive patients. The result of this study showed that adult HIV-positive patients with good and poor ART adherence have a positive effect on the mean changes in CD4 count compared to patients with fair adherence. This finding was similar to a study conducted by [15].

Our study showed that WHO clinical stage-II patients have a lower risk of death than clinical Stage-I patients. This result is in agreement with a study done by [15–17], but the result of this study contradicts a study done by [14]. This might be due to the following reasons; the study area, sample size, and study period.

In patients who were bedridden when starting ART treatment, a mean change in the square root of the CD4 count change was 2.478 times higher than in ambulatory patients. A similarly reported study by [13] had a significant effect on the mean changes in CD4 count after patients began antiretroviral treatment. Also, according to the study done in Jimma by [18], based on the joint modeling of longitudinal CD4 count and time-to-death of HIV/TB co-infected patients, the functionality of bedriddiness had significant effects when compared to ambulatory.

The estimates of the association parameters in the joint analysis are significantly different from zero, confirming that the two sub-models are associated. This result is supported by a study done by [15, 19]. The negative value of the association parameter (-0.115) indicated that the slope of the square root of CD4 counts was negatively associated with the risk of death, and with a unit increase in the square root of the CD4 count, the risk of death decreased.

### Limitations and strength of the study

The study provided an overview of the globe through a variety of models and kinds. The variables that had a substantial effect on HIV patients in previous studies were related to this study, as well as compared and contrasted within it. However, the implementation of surviving patients was low ranks everywhere, except in the little body, which was a major issue for patients. Because of the body it may concern doesn’t care in this situation. Furthermore, there was no study in the pastoralist area that was relevant to this study.

## Conclusions

They concluded that there was an association between the mean changes in the square root of the CD4 count and the survival time of HIV patients using the joint model. Covariates such as visiting time, age, weight, educational status, ART adherence, functional status, WHO stage, place of residence, TB, family history, and opportunistic infection disease had a statistically significant effect on the joint models at the 5% level of significance.

Based on the results, the further study should focus on identifying the supporting reasons for factors that significantly affect the mean changes in CD4 cell count and survival time of patients by developing models and adding additional outcomes like viral CD4 count.

## Supporting information

S1 Fig Individual scaled Schoenfeld residual test plots. (JPG)

S2 Fig Normal Q-Q plot and histogram plots. (JPG)

S1 Data Data supporting conclusions of the manuscript. (CSV)

## References

[1] Joint United Nations Programme on HIV/AIDS, 2012. Global Report: UNAIDS Report on the Global AIDS Epidemic: 2012. UNAIDS, Geneva.

[2] Siraj M., Gedamu S., & Tegegne B. (2022). Predictors of Survival Time Among HIV-Infected Adults After Initiating Anti-Retroviral Therapy in Kombolcha Town: A 5-Year Retrospective Cohort Study. *HIV/AIDS-Research and Palliative Care*, 181–194. doi: 10.2147/HIV.S359495 35464618 PMC9020508

[3] Getaneh T, Dessie G, Desta M, Assemie MA, Alemu AA, Mihiret GT, et al. Early diagnosis, vertical transmission of HIV and its associated factors among exposed infants after implementation of the Option B+ regime in Ethiopia: a systematic review and meta-analysis. IJID regions. 2022 Sep 1; 4:66–74. doi: 10.1016/j.ijregi.2022.05.011 35813560 PMC9256659

[4] Lulseged S. Pediatric HIV epidemic: Status and prospects in Ethiopia. Ethiopian Journal of Pediatrics and Child Health. 2023 Aug 11;18(1).

[5] Wilhelmson S, Reepalu A, Tolera Balcha T, Jarso G, Björkman P. Retention in care among HIV-positive patients initiating second-line antiretroviral therapy: a retrospective study from an Ethiopian public hospital clinic. Global health action. 2016 Dec 1;9(1):29943. doi: 10.3402/gha.v9.29943 26765104 PMC4712321

[6] Alebel A., Engeda E. H., Kelkay M. M., Petrucka P., Kibret G. D., Wagnew F., et al. (2020). Mortality rate among HIV-positive children on ART in Northwest Ethiopia: a historical cohort study. *BMC Public Health*, 20, 1–11. 32854692 10.1186/s12889-020-09418-6 PMC7457276

[7] UNAIDS. (2022). Global AIDS Update: In Danger; July 2022. UNAIDS, AIDSinfo website; accessed July 2022, http://aidsinfo.unaids.org/. UNAIDS, 2022 Core epidemiology slides; July 2022. UNAIDS, Global HIV statistics 2022 fact sheet; July 2022; UNAIDS, UNAIDS data 2022; July 2022.

[8] Verbeke G, Molenberghs G, Verbeke G. Linear mixed models for longitudinal data. Springer New York; 1997.

[9] DR C. Regression models and life tables. JR Stat Soc. 1972; 34:248–75.

[10] West BT, Welch KB, Galecki AT. Linear mixed models: a practical guide using statistical software. Chapman and Hall/CRC; 2022 Jun 24.

[11] Rizopoulos D, Hatfield LA, Carlin BP, Takkenberg JJ. Combining dynamic predictions from joint models for longitudinal and time-to-event data using Bayesian model averaging. Journal of the American Statistical Association. 2014 Oct 2;109(508):1385–97.

[12] Seid A, Getie M, Birlie B, Getachew Y. Joint modeling of longitudinal CD4 cell counts and time-to-default from HAART treatment: a comparison of separate and joint models. Electronic Journal of Applied Statistical Analysis. 2014 Oct 14;7(2):292–314.

[13] Kebede MM, Zegeye DT, Zeleke BM. Predictors of CD4 count changes after initiation of antiretroviral treatment in University of Gondar Hospital, Gondar in Ethiopia. Clinical Research in HIV/AIDS. 2015;1(2):1–5.

[14] Anjullo BB, Teni DA. Linear mixed modeling of CD4 cell counts of HIV-infected children treated with antiretroviral therapy. Advances in Public Health. 2021 Jan 29; 2021:1–6.

[15] Tegegne AS, Ndlovu P, Zewotir T. Determinants of CD4 cell count change and time-to default from HAART; a comparison of separate and joint models. BMC infectious diseases. 2018 Dec; 18:1–1. 29703155 10.1186/s12879-018-3108-7 PMC5922030

[16] Agegnehu CD, Merid MW, Yenit MK. Predictors of anemia among adult HIV positive patients on first-line antiretroviral therapy in northwest Ethiopia: a retrospective follow-up study. HIV/AIDS-Research and Palliative Care. 2021 Apr 29:455–66. 10.2147/HIV.S280338 PMC8096420 33958896

[17] Afrashteh S, Fararouei M, Ghaem H, Aryaie M. Factors associated with baseline CD4 cell counts and advanced HIV disease among male and female HIV-positive patients in Iran: a retrospective cohort study. Journal of Tropical Medicine. 2022 Jul 7;2022. doi: 10.1155/2022/8423347 35846073 PMC9283081

[18] Temesgen A, Kebede T. Joint modeling of longitudinal CD4 count and weight measurements of HIV/tuberculosis co-infected patients at Jimma University specialized hospital. Annals of Data Science. 2016 Sep; 3:321–38.

[19] Tayu Nigusie A. Longitudinal Cd4 Cell Count and Time to Death Among HIV Infected Children Initiating Antiretroviral Therapy. *Unpublished Document*. 2019 July.

