# Supplementary figures and images for: Determinants of longitudinal changes of CD4 cell count and survival time to death of HIV/AIDS patients treated at Yabelo General Hospital, the case of pastoralist area: Using joint modelling approach
Source: PLoS One. 2024 Jun 24;19(6):e0305519. doi: 10.1371/journal.pone.0305519 (PMC11195975; doi:10.1371/journal.pone.0305519)

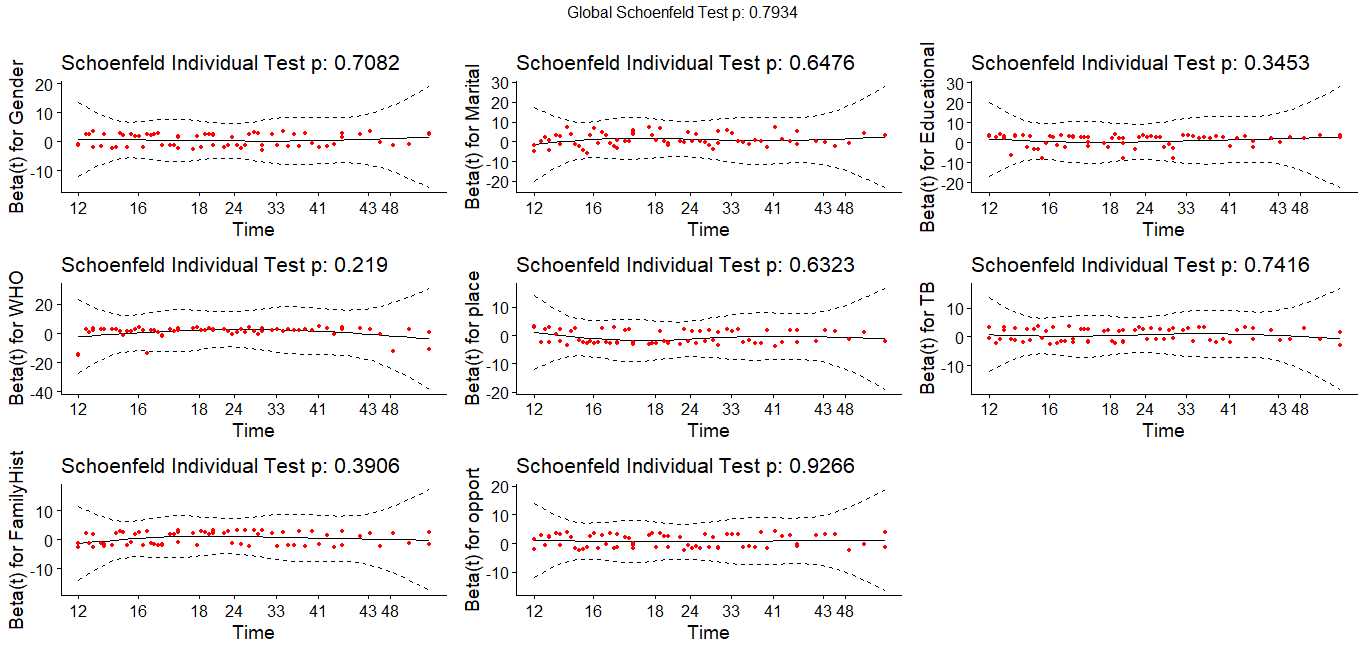

Supplement: S1 Fig — (JPG) [file pone.0305519.s001.jpg]

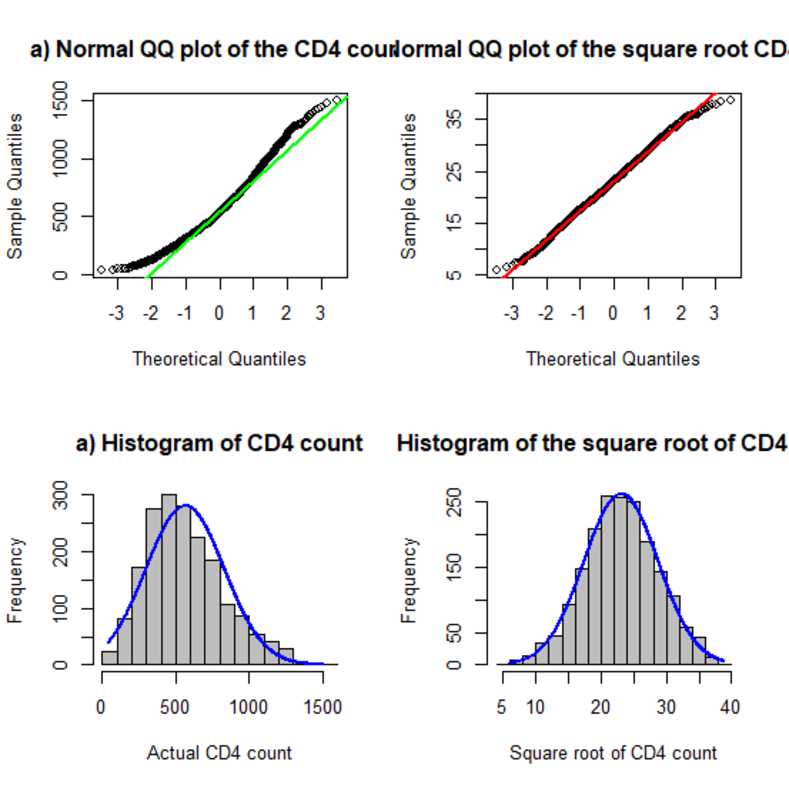

Supplement: S2 Fig — (JPG) [file pone.0305519.s002.jpg]
